# Familiarity with ChatGPT Features Modifies Expectations and Learning Outcomes of Dental Students

**DOI:** 10.1016/j.identj.2024.04.012

**Published:** 2024-04-26

**Authors:** Jelena Roganović

**Affiliations:** Department of Pharmacology in Dentistry, Faculty of Dental Medicine, University of Belgrade, Belgrade, Serbia

**Keywords:** Artificial intelligence, ChatGPT, Education dental, Ethics, Pharmacology

## Abstract

**Objectives:**

The number of approvals for AI-based systems is increasing rapidly, although AI clinical trial designs lack consideration of the impact of human–AI interaction. Aim of this work was to investigate how reading of an AI system (ChatGPT) features/descriptions could influence the willingness and expectations for use of this technology as well as dental students’ learning performance.

**Methods:**

Dental students (N = 104) were asked to learn about side effects of drugs used in dental practice via reading recommended literature or ChatGPT. Expectations towards ChatGPT were measured by survey, before and after reading of a system features description, whilst learning outcomes were evaluated via pharmacology quiz.

**Results:**

Students who used ChatGPT (YG group) showed better results on the pharmacology quiz than students who neither read the description nor employed ChatGPT for learning (NN condition). Moreover, students who read the description of ChatGPT features yet did not use it (NG) showed better results on the pharmacology quiz compared with the NN condition, although none of them employed ChatGPT for learning. The NG students compared to the YG students had less trust in AI system assistance in learning, and after the AI system description reading, their expectations changed significantly, showing an association with quiz scores.

**Conclusions:**

A majority of students in our cohort was reluctant to use ChatGPT. Furthermore, familarity (reading) with ChatGPT features appear to alter the expectations and enhance learning performance of students.suggesting an AI description–related cognitive bias. Hence the content description of ChatGPTshould be reviewed and verified prior to AI system use for educational purposes.

## Introduction

The number of applications for AI-based educational and related systems is increasing rapidly, although universal guidelines for evaluation of commercially available AI systems are not yet established. A recent study analysed the US Food and Drug Administration summaries of approved AI tools for medicine and found that almost all of the AI devices i) underwent only retrospective studies at their submission, ii) lacked validation with a side-by-side comparison of clinicians’ performances with and without AI, and iii) human–AI interactions were not been considered.^1^ In clinical trials in medicine/dentistry, presence of placebo was considered if the investigated drug/treatment was found effective only because study participants’ expectation in the drug/treatment efficacy, leading to a positive evaluation of it's effectiveness. These expectations of effectiveness are controlled via a nonactive substance/sham treatment provided to participants who are led to believe, via blinding, that the substance/treatment is active and will have an effect. However, in an investigation of AI system performance, participants cannot be blinded and are aware of whether they are using an AI system; thus, the question arises of whether there is a placebo effect that could cloud AI clinical trial results. A recent study by Kosch et al^2^ investigated the placebo effect of AI in human–computer interaction and concluded that a placebo effect could be present in human–AI interactions, due to the belief that AI system functionality although the participants were actually employing a sham treatment protocol. Thus, the AI technology altered expectations related to the system description, suggesting that the description of the AI system could bias expectations. In the area of dentistry, Mertens et al^3^ showed that using AI-based software for caries detection was followed by enhanced diagnostic accuracy on the part of dentists. However, in the same study, the authors noted a large discrepancy between the diagnostic performance (sensitivity) of dentists who did not use AI software in 2 diagnostic studies: The dentists who did not use an AI system exhibited greater diagnostic accuracy (sensitivity) in the study by Martens et al^3^ compared with a similar study by Cantu et al^4^ (0.72 and 0.36, respectively). In addition to selection bias, an underlying reason might be the use of a different study protocol: In the study by Martens et al,^3^ even if dentists did not use AI, they were familiar with an AI-based software handbook (description), whilst that did not seem to be the case in the study by Cantu et al.^4^ Thus, the question arises of whether technology system description–altered expectations may enhance one's performance even if technology (real or sham) was not actually employed. In addition, what implications might this have on AI clinical trial design and evaluation? Thus, aims of this work were to investigate how the reading of ChatGPT (OpenAI) features descriptions influence willingness and expectations for use of this technology as well as dental students’ learning performance.

## Method

**Fig. 1:**
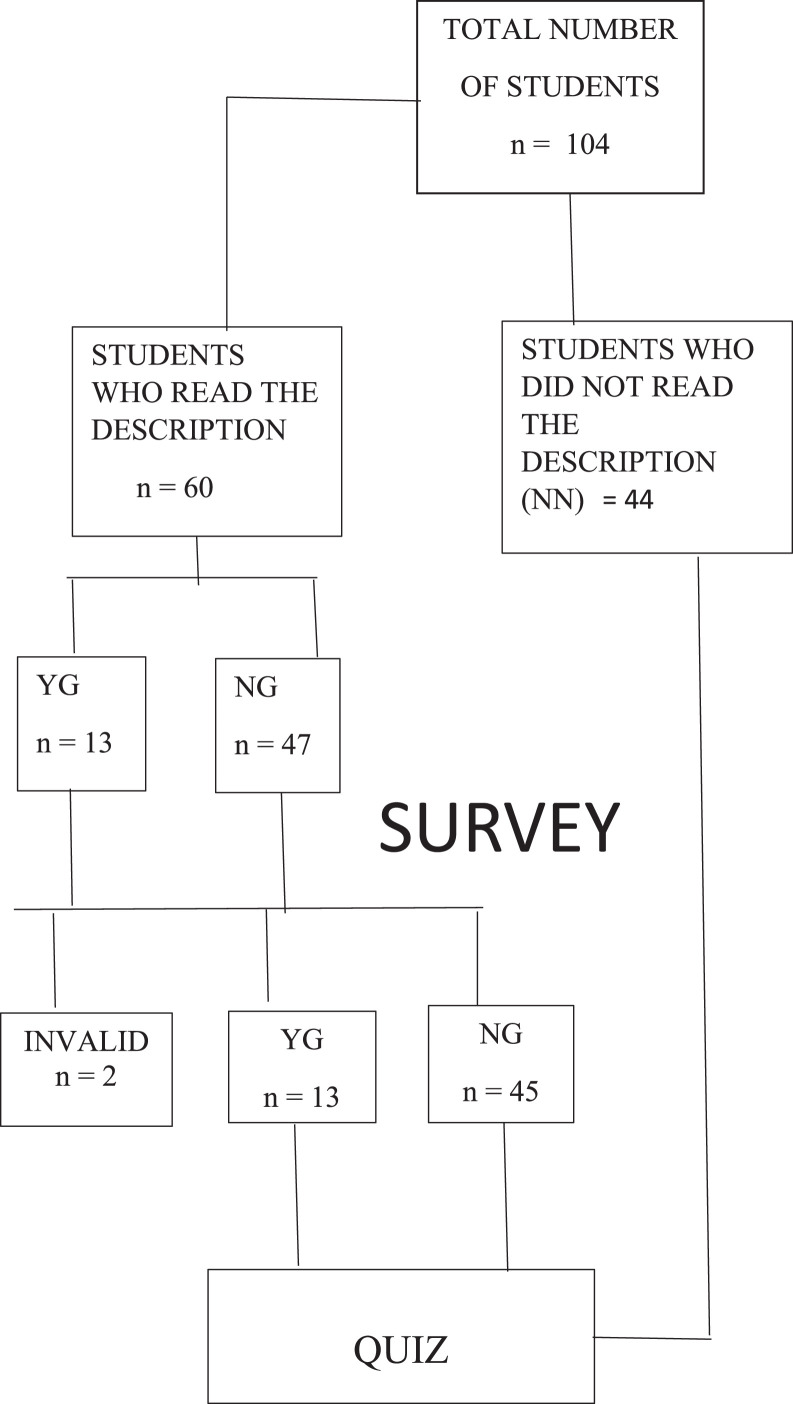
Study flowchart. YG, used ChatGPT; NG, did not use ChatGPT, but read its description; NN, neither used ChatGPT nor read the description.

Total of 104 (22 male and 82 female) third-year dental students attending a pharmacology course were included in the study. Ethical approval was obtained from the Ethics Committee of the Faculty of Dentistry, number 36/50, 2023. Students were asked to learn and then write an essay on the side effects of drugs used in dental practice, by using usual literature (a pharmacology book)^5^ or an AI-based language processing tool that is freely available online—ChatGPT-3.5—after they voluntary decided which condition they would be in. Groups were as follows: YG, “yes” group (will use ChatGPT); NG, “no” group (will not use ChatGPT but will read its description); or NN, “none” group (will neither use ChatGPT nor read the description). Before reading the ChatGPT features description, only 13 participants were willing to be in the YG group, whilst 47 decided to be in the NG group and 44 in the NN condition. Interest in using ChatGPT as well as attitudes and expectations regarding this AI technology were measured in the YG and NG groups by survey, before and after reading a ChatGPT description (Figure 1). On the same day, all students completed an 11-item questionnaire with closed-ended questions in the Serbian language that was prepared based on a review of the literature assessing expectations and acceptance of AI technology,^2^^,^^6^ under the Unified Theory of Acceptance and Use of Technology (UTAUT) framework, a validated model used to analyse technology acceptance in a Serbian sample^7^ adapted to the objectives of the present study. Thus, the survey aimed to score, amongst others things, performance expectancy (“using ChatGPT would help me to write an essay better or faster, and it is useful”), effort expectancy (“I find ChatGPT easy to use”), or attitude (“Using ChatGPT is wise”). A 5-point Likert scale was used to measure attitudes and expectations for use of this AI technology, and mean sums of scores were obtained. The students rated statements from 1 (*strongly disagree*) to 5 (*strongly agree*); the higher the score, the higher degree of agreement.

The description of ChatGPT was taken from the OpenAI official website (https://help.openai.com/en/articles/6783457-what-is-chatgpt), with an added sentence with pattern of pharmacology questions they could discuss with ChatGPT. In order to measure task (learning) performance, all involved students took a quiz (7 questions completely covered by recommended literature [pharmacology book^5^]), which was graded by a teaching assistant who was blinded to group assignment. Data were managed by GraphPad Prism software v.10. Results were expressed as mean score ± SD. Repeated-measures *t* test was applied to analyse students’ attitudes and expectations prior to and after reading the description. One-way analysis of variance with Bonferroni post hoc test was applied to compare score results amongst the 3 groups. Multiple linear regression was employed to investigate which expectations affect learning performance (quiz scores). A *P* value ˂.05 was considered significant.

## Results

### Survey scores before reading the description

**Fig. 2:**
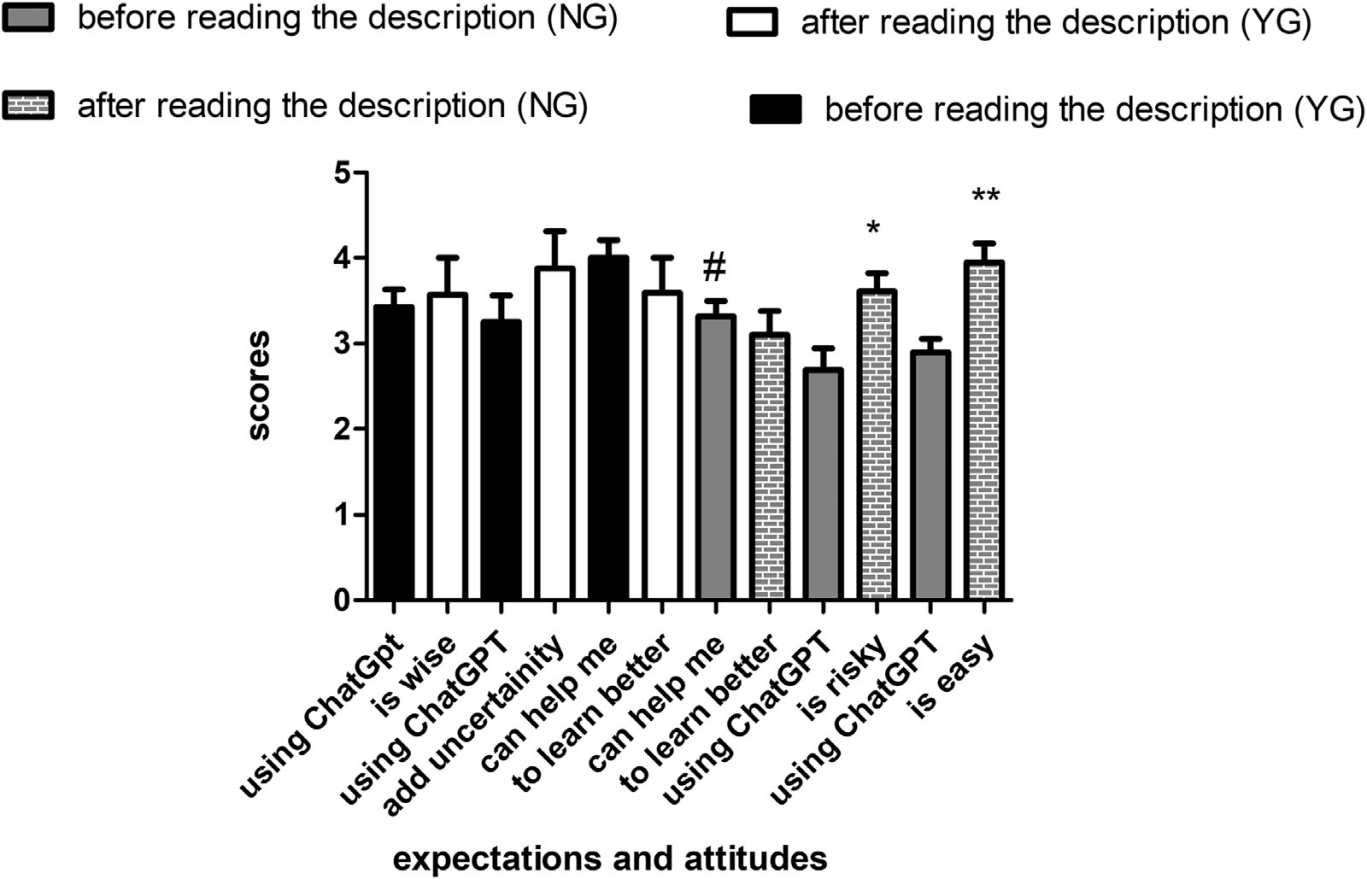
Expectations and attitudes towards ChatGPT before and after reading its description in NG and YG groups. *P ˂ .05. **P ˂ .01 before vs after reading the description. #*P* ˂ .05 NG vs YG. YG, used ChatGPT; NG, did not use ChatGPT, but read its description; NN, neither used ChatGPT nor read the description.

Choosing to participate in the study as well as to use an AI-based tool in learning was voluntary. The fact that only 13 of the students initially (12.5%) were willing to use AI in learning is important to consider. The mean survey score of expectations and attitudes towards ChatGPT was higher in the YG compared to the NG group (44.0 ± 4.7 vs 34.5 ± 8.1; *P* ˂ .05). Performance expectations were significantly different between YG and NG groups, with participants in the YG group expecting that ChatGPT would help them to learn and write better (4.0 ± 0.2 vs 3.3 ± 0.1; *P* ˂ .05). There was no statistical difference in scores amongst groups when comparing AI-associated risk assessment (3.3 ± 0.2 vs 2.7 ± 0.2; *P* > .05; Figure 2).

### Survey scores after the reading of the description

Of 60 survey responses, 2 were invalid and in 5 cases (8.3%) there were no changes in responses before and after reading the description. After reading the description, 3 students changed their mind and entered the YG group; at the same time, 2 students initially in the YG group switched to the NG group after they read the description. After reading the ChatGPT features' description, the NG group was the one with the highest number of participants to change their scores regarding effort expectancy (expecting that ChatGPT is easier to use, 2.9 ± 0.1 before reading vs 3.9 ± 0.2 after reading; *P* ˂ .001), overall risk (using ChatGPT is riskier, 2.7 ± 0.2 before reading vs 3.6 ± 0.2 after reading; *P* ˂ .01), and performance expectancy (using ChatGPT would help me to write an essay better, 3.3 ± 0.1 before reading vs 3.1 ± 0.2 after reading; *P* > .05; Figure 2). After reading the description, the YG group was the one with the highest number of participants to change their scores regarding performance expectancy (using ChatGPT would help me to write an essay better, 4.0 ± 0.2 before reading vs 3.6 ± 0.4 after reading; *P* > .05; Figure 2).

### Pretest on ChatGPT accuracy on drug side effects questions

Due to uncertainty surrounding the ChatGPT model's training dataset regarding information that the system has about drugs side effects, the pretest was conducted and ChatGPT's accuracy questions regarding drug side effects was 89%. Incorrect answers were due to a linguistic problem: When the questions were written in English, the responses were correct (eg, What drug does not induce xerostomia: aspirin, promethazine, or pilocarpine? What drugs may induce cleft lip and palate in newborns: antihistamines, beta blockers, antibiotics, or anticonvulsant drugs?).

### Number of correct responses on the quiz

**Fig. 3:**
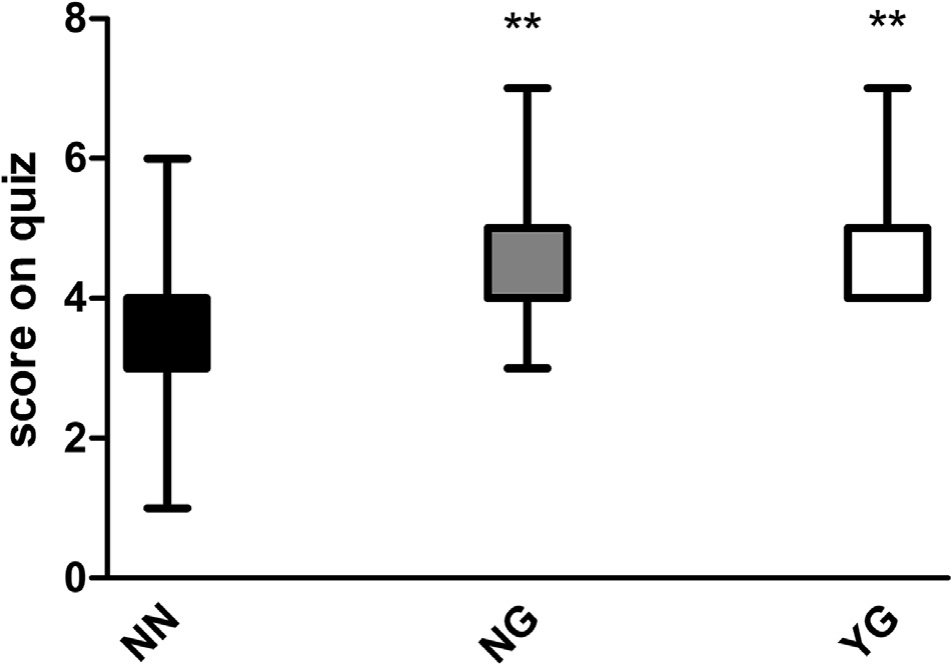
Pharmacology quiz scores in NG, NN, and YG groups. **P ˂ .01 vs NN condition. YG, used ChatGPT; NG, did not use ChatGPT, but read its description; NN, neither used ChatGPT nor read the description.

There was a significant difference in the number of correct responses between the NN and NG groups (3.61 ± 1.20 vs 4.65 ± 0.91; *P* < .001) as well as between the NN and YG groups (3.61 ± 1.26 vs 4.85 ± 0.89; *P* < .01), whilst such a difference was not observed between the NG and YG groups (4.65 ± 0.91 vs 4.85 ± 0.89; *P* > .05; Figure 3). Due to the voluntary nature of the study, post hoc achieved power of the study was calculated and a total number of 88 participants (44 per group) was determined to be able to demonstrate a medium difference (effect size, 0.5) in quiz scores between NN and NG groups for a test power of 85% and alpha probability of 0.1. Calculations were performed using a *t* test for independent groups (statistical software package G*Power 3.1).

### Relationship between expectations and quiz scores in NG and YG groups

**Fig. 4:**
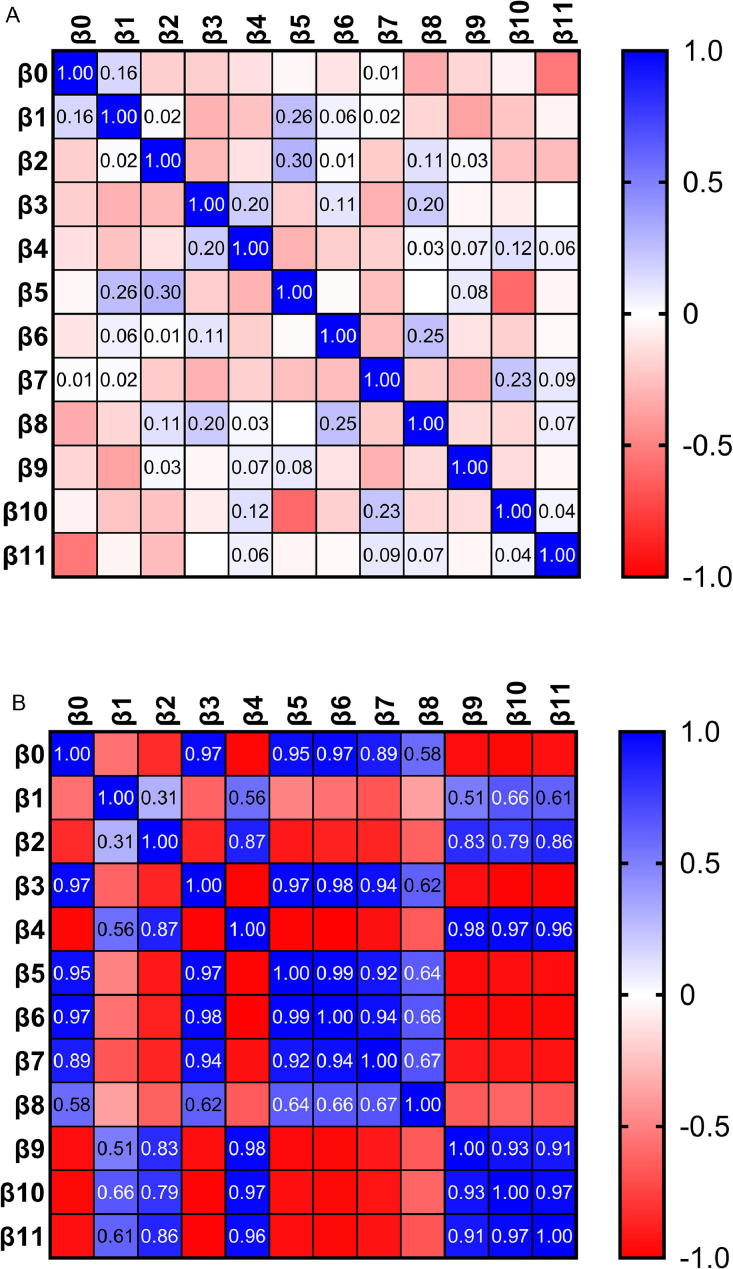
The magnitude of covariance between variables (expectations) in NG (**A**) and YG (**B**) conditions. The higher absolute magnitude of covariance suggests a stronger relationship between variables, whilst the sign indicates the direction of the relationship between variables. YG, used ChatGPT; NG, did not use ChatGPT, but read its description.

Multiple linear regression was employed to investigate which expectations (attitudes) affect learning performance (quiz scores) in the NG group, and results are shown in Table 1. Expectations of effort (“using ChatGPT is easy”), novelty (“using ChatGPT is a new experience”), and performance (“using ChatGPT is useful”) were significantly related to quiz scores, with an inverse relationship found between performance expectations and quiz scores. The relationship between variables (expectations), represented as the covariance matrix between parameters, is presented in Figure 4A, whilst low levels of multicollinearity were obtained, represented by a variance inflation factor (VIF) <5 (Table 1). No significant relationships were found between expectations and quiz scores in the YG group, whilst a high magnitude of the covariance between parameters (Figure 4B) with a VIF > 10 was observed (Table 2), suggesting high levels of multicollinearity.

**Table 1:** Relationship between expectations and quiz scores and multicollinearity in the NG group obtained by multilinear regression.

Parameter estimates	Variable	Coefficient (estimate)	Standard error	95% CI (asymptotic)	|t|	*P* value	VIF
β_0_	Intercept	2.42	0.82	0.74 to 4.10	2.93	.006	
β_1_	Can help to learn better	−0.03	0.14	−0.33 to 0.26	0.24	.80	2.04
β_2_	Using ChatGPT could be risky	0.07	0.16	−0.25 to 0.41	0.48	.63	1.58
β_3_	Using ChatGPT is easy	0.52	0.16	0.18 to 0.87	3.12	.003	2.05
β_4_	I intend to use ChatGPT	0.13	0.12	−0.11 to 0.37	1.06	.29	1.48
β_5_	I am interested in new technologies	0.16	0.14	−0.13 to 0.46	1.10	.27	2.35
β_6_	Using ChatGPT is new experience	0.25	0.11	0.02 to 0.48	2.24	.03	1.47
β_7_	Using ChatGPT is useful	−0.40	0.18	−0.78 to −0.03	2.20	.03	2.40
β_8_	Using ChatGPT is wise	0.18	0.12	−0.07 to 0.44	1.47	.15	1.33
β_9_	Can help me to learn more quickly	0.16	0.17	−0.18 to 0.52	0.96	.34	2.07
β_10_	Using ChatGPT is fun	−0.26	0.18	−0.63 to 0.09	1.48	.14	2.34
β_11_	Add great uncertainty to my interaction	−0.11	0.14	−0.40 to 0.17	0.81	.42	1.11

VIF is used to detect multicollinearity amongst the independent variables. VIF values <5 suggest low levels of multicollinearity.

NG, did not use ChatGPT, but read its description; VIF, variance inflation factor.

**Table 2:** Relationship between expectations and quiz scores and multicollinearity in the YG group obtained by multilinear regression.

Parameter estimates	Variable	Coefficient (estimate)	Standard error	95% CI (asymptotic)	|t|	*P* value	VIF
β_0_	Intercept	17.76	15.78	−182.8 to 218.3	1.125	.4625	
β_1_	I am interested in new technologies	−0.22	0.64	−8.42 to 7.98	0.34	.79	5.34
β_2_	Using ChatGPT is new experience	−1.62	2.48	−33.24 to 29.98	0.65	.63	21.37
β_3_	Using ChatGPT is useful	4.08	6.27	−75.65 to 83.83	0.65	.63	368.5
β_4_	Using ChatGPT is wise	−9.77	14.85	−198.5 to 179.0	0.65	.62	2040.0
β_5_	Can help me to learn more quickly	2.02	4.41	−54.05 to 58.10	0.45	.72	123.7
β_6_	Can help to learn better	5.374	7.45	−89.35 to 100.1	0.72	.60	606.7
β_7_	Using ChatGPT is easy	1.27	2.09	−25.40 to 27.95	0.60	.65	22.58
β_8_	Using ChatGPT is fun	0.76	0.69	−8.11 to 9.64	1.09	.47	3.42
β_9_	Using ChatGPT could be risky	−1.85	2.64	−35.43 to 31.72	0.70	.60	82.45
β_10_	Add great uncertainty to my interaction	−1.95	3.05	−40.79 to 36.88	0.63	.63	110.3
β_11_	I intend to use ChatGPT	−3.15	4.42	−59.34 to 53.04	0.71	.60	152.5

YG, used ChatGPT; VIF, variance inflation factor.

## Discussion

Present results suggest that a small number of students were interested in employing ChatGPT in learning, mainly because they doubted its performance abilities, safety, and reliability, and this finding is in the line with a previous investigation of AI acceptance by dental students.^8^ Despite a small number of participants, the YG group showed significantly better quiz results than the NN condition, whilst the difference in learning performance of the YG group compared with the NG group was not obtained, nor was the association of expectations with learning performance of YG participants, which has previously been suggested to represent placebo.^2^ Moreover, beside a small number of participants, regression analysis of YG group data revealed high multicollinearity. Thus, a high correlation between variables (expectations) represents difficulty in interpreting the effect of each expectation on task performance separately, because expectations' effects were confounded by their correlation. Due to these limitations, the exact contribution of ChatGPT to learning task accomplishment could not be established. Thus, focus in this research was on investigating how reading ChatGPT features descriptions affected students’ quiz performance, since post hoc analysis revealed that differences observed between NG and NN conditions in quiz scores could be considered reliable.

Doubtless, the reading of ChatGPT features descriptions altered expectations and attitudes towards this technology and was associated with enhanced quiz performance in participants who read the description but did not use the AI system. Understanding how descriptions of the novel technology influence task performance and might induce cognitive bias or placebo effects is important for standardisation of clinical trials involving evaluation of AI technology.

A placebo effect has already been recognised in human–AI interaction: manipulating AI system descriptions manifest subjectively in enhanced performance expectations which persisted after the use of the AI system, while a relationship was found between performance expectations and participants' task performance.^2^ In the present study, ChatGPT was used as an AI-based model for investigating AI system description–associated expectations and their relation to students’ performance on a pharmacology quiz. Presently, reading of the ChatGPT features description has been shown to alter expectations of 92% of dental students, as measured by survey scores. However, it did not increase expectations towards belief in superior AI system support. On the other side, effort expectations (“ChatGPT is easy to use”) and estimations of risks associated with its use (“use of ChatGPT is risky”) were significantly changed after the reading of the description in the NG group. Moreover, better pharmacology quiz scores were observed in the group of students who read the description compared to those who did not read the description, although none of them used ChatGPT for learning. These scores were associated with altered performance, effort, and novelty expectations in this group.

The quiz covered the theme of side effects of drugs used in dentistry, with course material found within a pharmacology book^5^ that was recommended literature for preparing for the quiz. Better results presently obtained in the NG group could be related to AI system features descriptions for several reasons: Expectation scores were significantly associated with quiz scores in the NG group, and other factors—such as selection bias and greater ambition and interest in the NG group compared with the NN condition—could not be excluded. Regarding possible explanations of the relation between students’ altered expectations and learning performance, psychology research indicates that one's perceptions and decisions may be influenced by cognitive biases or implemented through nuanced cues in the system description. In addition, minor differences in initial instructions could have a major impact on task performance.^9^ Thus, a possible explanation of the association between reading the AI system features' description and enhanced task performance could be the adoption of the described performance pattern utilised by AI (and explained in the AI system description) which results in altered participants’ perspectives regarding the problem and enhanced task performance. Presently, a description of the pharmacology question pattern was added (eg, “side effects of antibiotics are …”; “the most frequent side effect of NSAIDs is …”) to the description of ChatGPT features taken from the official site, and this was later applied to the quiz questions. Adoption of the pharmacology question pattern outlined in the description could have influenced a better result on the quiz in the NG group, in which students read the description. Students in the NN condition learned from the book, in which a different approach to the pharmacologic subject had been applied (eg, “intraoral side effects were induced by various drugs …”; “side effects in GIT include … and are induced by following drugs …”), although the book was considered to contain the same amount of information as the AI system description.

Future research is needed to investigate the psychological aspects associated with AI technology acceptance and task performance, but for now it seems that introducing AI system features descriptions for all participants in the study would not be ethically problematic. Limitations of this study are as follows: Due to the small number of participants who used ChatGPT in learning, regardless of significantly better quiz results, the exact contribution of ChatGPT in learning about drug side effects could not be established. However, the design employed enabled investigation of students’ interest in employing ChatGPT in learning and writing. Regardless of the pretest conducted, a precaution in data interpretation regarding representativeness of the ChatGPT model's training dataset should be emphasised. Also, it is probable that another reason distinct to AI system description reading could affect quiz performance , such as the one related to learning capacity, or ambition, or motivation. Nevertheless, present results showed that reading the description alone alters attitudes and expectations and may influence task performance.

Implications of the present results should be considered: If a placebo effect or cognitive bias result from AI system description reading, the objective performance of the AI system must be verified in the context of participants’ comprehension of this description. A “well-written” description of the AI system may induce a belief that AI has a better performance than it truly does and thus, users’ (health facility, dentist) perceptions of system superiority may be manipulated. From the safety and ethical perspectives, such an overestimation due to description manipulation could lead to a misuse of AI systems by health care practitioners as a primary instead of a supportive tool in clinical decision-making; use of autonomous systems, such as dental robots, are of special concern. On the other hand, explaining the functionality of AI systems to patients might be associated with improved compliance and better treatment outcomes, as has been shown in a previous clinical trial.^10^ Thus, AI system descriptions should be verified and approved by regulatory bodies.

## Conclusions

Dental students showed low interest for using ChatGPT in pharmacology learning and low expectations regarding ChatGPT performance, as well as doubts regarding its safety and reliability. At the same time, reading of ChatGPT features descriptions altered expectations and enhanced learning (quiz) performance in participants who did not use the AI system, which suggests AI system description–related cognitive bias. Thus, AI description content should be reviewed and verified prior to AI system use, whilst introduction of AI system features descriptions should be considered for all participants in AI clinical trials.

## Conflict of interest

None disclosed.
